# Does prism adaptation treatment reduce spatial neglect and improve function?

**DOI:** 10.3389/fresc.2025.1539887

**Published:** 2025-02-05

**Authors:** Peii Chen, Kimberly Hreha, Catrina MacPhee, Amber Salter, Gail A. Eskes

**Affiliations:** ^1^ Center for Stroke Rehabilitation Research, Kessler Foundation, West Orange, NJ, United States; ^2^ Department of Physical Medicine and Rehabilitation, New Jersey Medical School, Rutgers University, Newark, NJ, United States; ^3^ Department of Orthopaedic Surgery, Occupational Therapy Division, School of Medicine, Duke University, Durham, NC, United States; ^4^ Department of Psychology & Neuroscience, Dalhousie University, Halifax, NS, Canada; ^5^ Department of Neurology, University of Texas Southwestern Medical Center, Dallas, TX, United States; ^6^ Departments of Psychiatry and Psychology & Neuroscience, Dalhousie University, Halifax, NS, Canada

**Keywords:** efficacy, effectiveness, behavioral intervention, therapeutic treatment, rehabilitative therapy, spatial exploration, sensorimotor adaptation, unilateral spatial neglect

## Abstract

The potential of using prism adaptation for treating spatial neglect (SN) was questioned when recent meta-analyses found inconsistent evidence. However, analyses of clinical datasets support the use of prism adaptation treatment (PAT) in reducing SN and improving function. The main objective of this review is to evaluate the current state of the evidence of PAT therapeutic effects, identify knowledge gaps, and make suggestions to guide further research and support clinical decision-making. We used the framework of the National Institutes of Health (NIH) Stage Model for Behavioral Intervention Development which provides guidance on best practices for developing effective behavioral interventions that can be implemented in real-world settings. This model emphasizes the interplay between mechanisms underlying therapeutic effects (“who” should receive the treatment and “how” best does it work?) and considerations of adaptability and feasibility in real-world settings. The present critical review led to the following conclusion: the use of the NIH Stage Model reveals the heterogeneity of PAT studies and challenges in advancing PAT as an effective intervention. The key mechanisms such as prism strength, treatment intensity, arm visibility and activities during treatment, and evaluation methods lack consensus. Therefore, clinical research teams must continue to collect evidence to determine critical mechanisms and the optimal protocol. Further research identifying the optimal PAT protocol is needed before another meta-analysis on PAT's clinical efficacy should be conducted again.

## Introduction

Spatial neglect (SN) is a disorder characterized by difficulties with reporting, responding, or orienting to information presented or mentally represented on the contralesional side of space (1–4). SN is caused by damage to neural networks critical to spatial processing and attention control (5–8), affecting multiple perceptual modalities (9–12) and thus multiple cognitive and motor functions (4, 13–16). Symptom presentations of SN are independent of primary sensory or motor defects (2, 17, 18). For example, conjugate eye deviation toward the ipsilesional side of space at rest (19, 20) and gaze preference toward the ipsilesional side during visual exploration, independent of vision or the passive range of motion of the eyes, are hallmark signs of SN (14, 21). While symptoms in the visual modality are most observable and reported, symptoms in the auditory, tactile, and proprioceptive modalities have been documented and can be disabling as well (9–12, 22, 23).

In addition to the presence of SN in different modalities, there is much heterogeneity in specific symptom presentation. Individuals with SN show substantial bias toward the ipsilesional side of space while appearing to “neglect” the contralesional side of space, although some implicit processing can be confirmed with certain tasks (24). The ipsilesional bias can be based on either egocentric (in relation to the person) or allocentric (in relation to an object in any spatial location) frames of reference (25, 26); for example, persons with left-sided egocentric SN may fail to locate objects in the space to or on the left side of their body, and those with left-sided allocentric SN may be unable to detect information appearing in the space to or on the left side of individual objects regardless where the objects are located in the left or right side of the person’s body. Symptoms also can be manifested in spatial regions on or of the body (i.e., personal space), within arms' reach (peri-personal space), and/or beyond arms' reach (extra-personal space) (27–29). Furthermore, not only the external space but also the mental space is affected; individuals with SN have difficulties retrieving information represented in the contralesional side of mental space (16, 30, 31). Finally, adding to the variability in SN symptom presentations, these symptoms can fluctuate depending on the internal mental capacity (e.g., fatigue level; focused vs. dual-tasking) (32, 33) and task requirement (e.g., erasing vs. marking targets; searching for vs. arranging objects; reporting vs. marking the center of a horizontal line) (34–36). Anosognosia for SN is also part of the syndrome, and a majority of patients with SN have poor self-awareness of their symptoms, overestimate their performance in tasks that require spatial ability (37), and thus may be unable to fully engage in therapy activities of strategy training (38).

There is currently no gold standard for how to screen, assess, or diagnose SN (39–41). According to a scoping review performed in 2018 (42), 292 SN tests (spanning across impairment and functional levels) have been published with the literature growing since then. This growing collection of measures reflects, at least in part, the challenges of measuring SN due to the heterogeneity of the disorder as well as the unpredictable variability in symptom presentations as described above. In other words, patients may show SN symptoms on certain tests but not all tests (43, 44), and the estimated incidence of SN varies depending on diagnostic methods (45), creating large variability in our understanding of its prevalence and long-term recovery (45).

Two common outcome measures have tried to account for this heterogeneity by including multiple tests—the conventional subtests of the behavioral inattention test (BIT-c) with six paper-based tests (viz., line crossing, star cancellation, letter cancellation, figure copying, line bisection, and free drawing) (46, 47) and the Catherine Bergego scale (CBS) (48) on its original questionnaire format or using a standardized procedure through Kessler Foundation neglect assessment process (KF-NAP) (49). BIT-c measures SN at the impairment level in the peri-personal space. CBS measures the impact of SN severity at the functional level in a range of daily activities including gaze orientation, limb awareness, auditory attention, personal belongings, dressing, grooming, navigation, collisions, meals, and cleaning after meals. With all things considered (e.g., diagnostic methods, injured cerebral hemisphere, and neglected side of space), overall SN occurs in approximately 30% of individuals who have had a unilateral stroke within the first 3 months (45). One-third of these individuals continue experiencing SN symptoms at the chronic stage when spontaneous improvement is highly unlikely (6, 26, 50, 51).

There are a variety of non-pharmaceutical treatments for SN (52–54), such as prism adaptation treatment (PAT), optokinetic stimulation combined with smooth pursuit (55, 56), visual scanning training (57), limb activation (58), visual imagery training (59), hemifield eye patching (60), neck vibration (61), vestibular stimulation (41), and transcranial magnetic stimulation (62). Among them, PAT is considered a promising option (63, 64). It is important to note that prism adaptation had been documented for decades before it was applied to treating SN. In his study published in 1963 (65), Harris asked participants, with unspecified neurological backgrounds, to point to a central visual target 90 times while wearing wedged prism lenses that shifted the visual field to the left or right by approximately 11°. Harris then observed aftereffects after prism removal in different pointing tasks, including pointing to visual targets without feedback (i.e., without seeing their upper limb) and with eyes closed, pointing to auditory targets, or simply pointing straight ahead (65). The aftereffects consisted of participants making pointing errors that were now biased toward the side of space opposite to the visual shift that had been induced by prism lenses. For example, after adapting to rightward-shifting prisms, when participants removed the prisms then leftward aftereffects occurred. Illustrations of prism adaptation and its aftereffects can be found in several published articles (66–70). Many replicated Harris's observations and expanded on the basic mechanisms of prism adaptation in healthy individuals (71–75). Then in 1998, a study led by Rossetti was published (67), which demonstrated prism adaptation as a potential treatment for SN. In the study, participants with left-sided SN made 50 pointing movements to visual targets while wearing wedged prism lenses that shifted the visual field to the right by 10° (experimental condition) or flat lenses that induced no visual displacement (sham-control condition). Similar to those reported by Harris (65), leftward aftereffects (i.e., increased pointing toward the left) were now observed after prism removal. More importantly, Rossetti et al. also observed changes in neuropsychological tests sensitive to SN symptoms, showing a reduction of left-sided SN symptoms immediately after prism removal and 2 h later (67). Rossetti et al. were the first to demonstrate the therapeutic effects of prism adaptation on SN. This led to the development of the treatment, i.e., PAT.

In general, PAT requires individuals with SN to complete a brief, repetitive arm reaching visual-guided exercise while temporarily wearing prism lenses that shift the visual field toward the egocentric ipsilesional side of space. Once the goggles are removed, the aftereffects from prism exposure induce an opposite bias in the contralesional direction. The therapeutic effect of PAT is hypothesized to be generated through implicit sensorimotor adaptation training, requiring no development of explicit strategies or new skills, and the benefits can be multimodal and across domains beyond sensorimotor activities (76–78). Interestingly, the potential of using PAT for treating SN was questioned by recent meta-analyses (53, 79–81) but supported by recent analyses of clinical datasets (82–86). Data generated from prospective clinical trials and data retrospectively extracted from clinical quality improvement practices are fundamentally different, and analysis methods are different too. Nonetheless, conflicting conclusions—both for and against the clinical implementation of PAT—are concerning or at least confusing for clinicians who rely on published evidence to guide their practice. In this critical synthesis and narrative review, we aim to achieve three objectives: to evaluate the current state of the evidence of PAT therapeutic effects based on recently published meta-analyses and clinical data analyses; identify knowledge gaps through critical comments; make suggestions to guide further research and to support clinical decision making based on the current status of PAT research findings. In particular, much of our efforts in this critical review are guided through the US National Institutes of Health (NIH) Stage Model for Behavioral Intervention Development (87).

### Does prism adaptation treatment work?

The groundbreaking study by Rossetti et al. (67) continues to inspire research and development of PAT for SN over the last 26 years, and a number of systematic reviews using meta-analysis methods have been published in recent years to seek evidence of PAT therapeutic effects. The latest 2021 Cochrane review on SN treatments, led by Longley (53), analyzed findings of PAT in eight RCTs of 257 participants and found no evidence for short- or longer-term therapeutic effects at either the SN-related impairment or functional level. Also published in 2021 were two other meta-analyses: Qiu et al. (80) found null effects in seven RCTs of 211 participants on the BIT-c or CBS when tested immediately or in the long term. In contrast, Li et al. (79) reviewed eight studies (244 participants) and reported on a series of meta-analyses of six studies that PAT resulted in benefits on the BIT-c or star cancellation test in the short term, while no effect was found for CBS. More recently, Szekely et al. (81) conducted a systematic review of published work up to June 2021 and a series of meta-analyses and found no evidence for PAT reducing SN measured using BIT-c (16 studies, 430 participants) or CBS (8 studies, 250 participants) immediately after treatment. While their broad criteria led to notable variability in the included patient characteristics or treatment protocols, including number of sessions (ranging from 1 to 20) and prism strength (5–17° of visual angle shifted by prisms), Szekely et al. found no relationship between these variables and treatment effect sizes. Thus, while acknowledging their conclusions were limited by the lack of coherence of the studies and the need for a more standardized approach in future work with well-controlled trials and sufficient sample sizes, Szekely et al. (81) suggested that since no short-term benefits were found, it was unlikely that long-term benefits would exist. Indeed, while the lack of well-controlled designs and sufficient sample sizes are important issues underlying negative results, the lack of consensus on PAT mechanisms and therapeutic protocol among the studies are also critical issues that make conclusions from meta-analyses problematic, i.e., the lack of evidence should not be interpreted as lack of effect. This logic is ignored in the conclusion by Szekely's emphasis on lack of benefit, rather than highlighting the need for better research methodology and standard therapeutic protocols to obtain stronger evidence.

Inconsistent conclusions are also highlighted through positive results from other evidence-gathering approaches. While RCTs and meta-analyses are regarded as a high level of evidence, real-world evidence of a treatment's use is also crucial for assessing the beneficial impacts of the treatment. An RCT is designed and conducted with a list of inclusion and exclusion criteria, controlled delivery of the intervention, and strict manualized protocols. However, in translating these findings to clinical care, therapy is individualized according to patient symptoms and goals of rehabilitation, and treatment is conducted within a multidisciplinary environment. The ultimate goal of developing PAT is to integrate it into regular clinical care, and analyzing large-scale clinical data is a necessary step toward this goal.

The latest real-world evidence of PAT therapeutic effects, based on the largest sample size reported to date, was part of a quality improvement initiative that implemented PAT for the treatment of SN conducted from June 2017 to March 2021 in 16 rehabilitation hospitals across 11 states in the United States (88). Occupational therapists were trained to use two standardized tools for assessing SN and using PAT in their regular clinical practice and, importantly, document the scores and usage. The assessment tool was KF-NAP (89, 90), and the treatment tool was Kessler Foundation prism adaptation treatment (KF-PAT) (49). KF-NAP provides standardized methods for functional evaluation of SN based on the CBS (as briefly mentioned above), and KF-PAT provides standardized procedures and equipment to administer and facilitate PAT. Once therapists finished training, they used the tools in their regular practice—thus, it was designed as a quality improvement project to improve clinical care by implementing standard assessment and treatment. At the end of the project (88), information available in medical records and therapist notes (i.e., clinical data) was analyzed retrospectively (84–86, 91, 92). A total of 4,454 individuals were assessed using KF-NAP, 82% of them were stroke survivors, and 55% of the total sample had SN (CBS > 0) within the first week admitted to rehabilitation hospitals (91).

Based on these clinical data, one analysis explored the beneficial impact of PAT on rehabilitation outcomes measured using the Functional Independence Measure (FIM), which was the most commonly used functional outcome measure in inpatient rehabilitation programs within the United States until 2019 (86). The analysis identified two groups of patients with SN (*n* = 156 per group) where one group received 8–12 sessions of PAT and the other did not receive PAT at certain sites. The two groups were matched in age, baseline severity in SN, and disability (measured using CBS and FIM at admission, respectively). The result showed a three-point difference in total FIM between groups (*η*^2^ = 0.035, *p* = 0.02), suggesting that PAT was associated with better rehabilitation outcomes (86). Another analysis of the clinical data (84) looked into treatment intensity and found that receiving more daily sessions of PAT was correlated with greater reduction of SN, as measured in CBS (*n* = 520; *b* = 0.16, SE = 0.06, *p* = 0.006) and greater improvement in FIM (*n* = 1720; *b* = 0.47, SE = 0.09, *p* < 0.001) after controlling for age, sex, type of brain injury, and relevant clinical characteristics. Moreover, patients who received eight or more PAT sessions showed greater CBS reduction as the frequency of PAT sessions increased (i.e., fewer days between two consecutive sessions). More PAT sessions also correlated with better FIM improvement in self-care, sphincter control, and transfers (84). Thus, this series of analyses provided practice-based evidence (93) supporting the use of PAT in inpatient rehabilitation settings.

### Answering “does PAT work?” through the NIH Stage Model for Behavioral Intervention Development

The terminology describing the effects of PAT is crucial in answering the question: “does PAT work?”. In the present article, “a therapeutic effect” is defined as a reduction of SN severity and/or symptoms after PAT, regardless of the presence of a control condition. A therapeutic effect can be at the impairment level (measured using neuropsychological tests) or functional level (measured using ecological assessments). When using “efficacy” and “effectiveness” to describe a therapeutic effect, a control condition must be included in the analysis. According to the US National Institutes of Health (NIH) definition, while both efficacy and effectiveness refer to the strength of a therapeutic effect, efficacy is used when the therapy is provided “under ideal and controlled circumstances” (94) while effectiveness is used “in clinical practice in the real world” (95).

**Figure 1:**
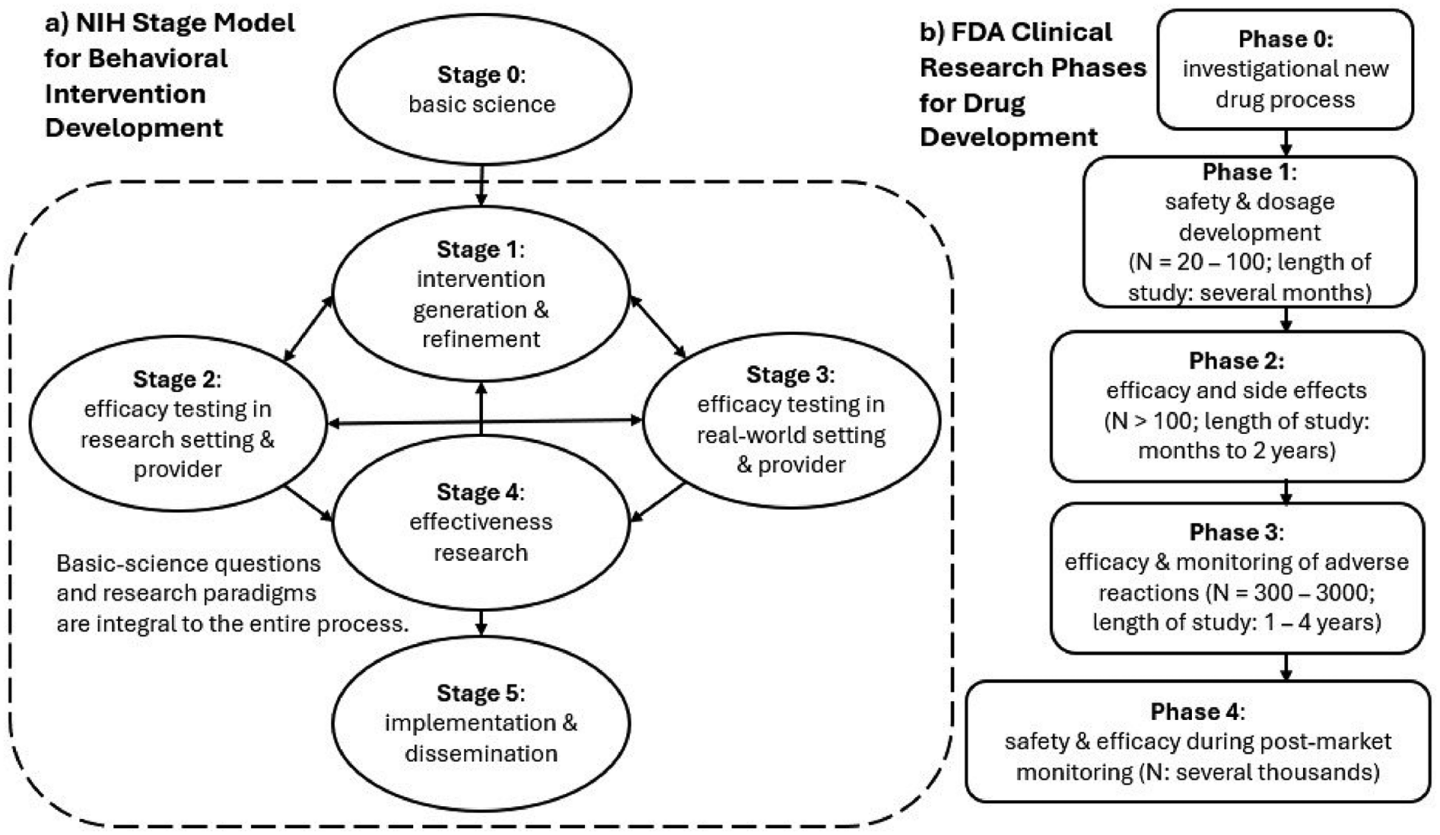
**(a)** NIH Stage Model for Behavioral Intervention Development vs. **(b)** FDA Clinical Research Phases for Drug Development. The NIH Stage Model emphasizes that questions of mechanisms of behavior change are relevant to every Stage. In addition, while stages are labeled from 1 to 5, the directions of progress are not linear from 1 to 5. The arrowheads can be bidirectional and point from different stages.

The goal of the present article is to address the question “does PAT work?” through a new perspective on the current state of PAT clinical research. This new perspective is derived from an NIH five-stage framework that provides guidance on best practices for developing effective behavioral interventions that can be implemented in real-world settings (Figure 1a) (87). Table 1 outlines the details of the stages. As emphasized in the NIH Stage Model, behavioral interventions are defined by the mechanisms governing their effects. Of note, the NIH Stage Model differs from the US Food and Drug Administration (FDA) Clinical Research Phases (96). The use of “phases” is ubiquitous in non-pharmaceutical clinical trials and has been criticized, and alternative approaches have been proposed (97, 98). Drug development is a linear process (Figure 1b), and developing a non-pharmaceutical behavioral intervention such as PAT, in contrast, is iterative and multidirectional (Figure 1a). Development of a behavioral intervention proceeds through an interplay between mechanisms underlying therapeutic effects (“who” should receive the treatment and “how” best does it work?) and considerations of adaptability and feasibility in real-world settings. Thus, defining and refining mechanisms are considered of both scientific *and* practical value in the NIH Stage Model. In addition, intervention development must also include validating materials (e.g., devices and therapist manuals) and methods (e.g., step-by-step procedures) used for administering the intervention; in other words, the fidelity of the planned intervention is crucial. The NIH Stage Model provides guidance on the goals of the research and who should be involved at the different stages, but the principles of the intervention should be a focus at each step. The usefulness of the NIH Stage Model has been reviewed (99, 100).

**Table 1:** NIH stage model for behavioral intervention development. Quotes are extracted directly from the NIH webpage.

Stage	NIH definition	Operational definition for PAT research
0	• “[B]asic science that occurs prior to intervention development, but is relevant (ultimately translatable) to intervention development.” • “Another type of basic science research—research on mechanisms of change—is an integral part of all other stages of intervention development.”	• Study with a control condition (randomization optional) • Seeking neural and sensorimotor mechanisms of prism adaptation in patients with SN through measurement of aftereffects and neuroimaging • Determining prism strength and visibility of arm movements during adaptation • Involving a single session for each of PAT and control conditions • Within- or between-participants condition manipulation
1	• “Creation and preliminary testing of a new behavioral intervention” including: a) Generating “new behavioral interventions as well as the modification, adaptation, or refinement of existing interventions (Stage 1A)” b) Culminating “in feasibility and pilot testing (Stage 1B)” • “Modification of an intervention for the purpose of making it more easily implementable.” • “Development of training materials” as “appropriate and necessary.” • “One can conduct Stage I studies in research settings, with research providers and research subjects; or in community settings with community providers or caregivers.”	• Study with a control condition (randomization optional) • Investigating treatment procedures such as visuomotor activities during prism adaptation for the purpose of enhancing feasibility and implementability • Involving multiple PAT sessions with a prospective or retrospective control group • Aiming to: a) Determine PAT methods or procedures. b) Explore PAT therapeutic effects but using outdated methods (such as low prism strength or treatment intensity). That is, a study might be at a higher stage originally, but it became Stage 1 as new evidence emerged. • Presence or absence of group-level comparisons in outcome measures
2	• “Pure efficacy” research • “[E]experimental testing of promising behavioral interventions in research settings, with research-based providers.”	• Randomized controlled trial • Involving multiple PAT sessions with latest evidence-supported methods^a^ • Aiming to explore therapeutic effects using non-ecological and/or ecological outcome measures specific to SN • PAT delivered in research or unspecified settings
3	• “Real-world Efficacy” research • “[E]xperimental testing of promising behavioral interventions in community settings, with community-based providers or caregivers, while maintaining a high level of control necessary to establish internal validity. Some refer to this as a hybrid (efficacy-effectiveness) stage.”	• Randomized controlled trial • Involving multiple PAT sessions with latest evidence-supported methods^a^ • Aiming to establish therapeutic effects through an ecological outcome measure specific to SN and beneficial impacts on overall function, mobility, quality of life, and other milestones of recovery • PAT delivered in clinical settings with considerable involvement and supervision of researchers during PAT delivery and data collection
4	• “Effectiveness” research • “[Examining] empirically supported behavioral interventions in community settings, with community-based providers or caregivers, while maximizing external validity.”	• Study with a control condition (randomization preferred but optional) • Involving multiple PAT sessions with latest evidence-supported methods^a^ • Aiming to establish therapeutic effects through an ecological outcome measure specific to SN and beneficial impacts on overall function, mobility, quality of life, community reintegration, employment, and other milestones of recovery • PAT delivered in clinical settings with limited or minimal involvement of researchers during PAT delivery and data collection
5	• “Implementation and Dissemination” research • “[Examining] strategies of implementation and adoption of empirically supported interventions in community settings.”	• Study with a control condition (randomization preferred but optional) • Involving multiple PAT sessions with latest evidence-supported methods^a^ • Aiming to determine the optimal strategy to implement the latest evidence-supported PAT regime • PAT delivered in clinical settings with minimal involvement of researchers during PAT delivery and data collection • Clinical leadership highly involved in facilitating, supporting, and supervising the implementation of the strategies

NIH, National Institutes of Health; PAT, prism adaptation treatment; SN, spatial neglect.

^a^ The latest evidence-supported methods when this article was written included (1) using high-strength prisms, e.g., 20 (or higher)-diopter lenses, or prisms that shift the visual field by 10 or greater degrees; (2) having patients complete at least three sessions a week and at least four sessions in total.

To reiterate, the NIH Stage Model highlights the consideration of the underlying mechanisms or principles of treatment to enhance the potency and implementation of the treatment. These principles focus on identifying “who” should benefit, “how” does treatment works, and the fidelity with which the treatment is applied. Keeping these principles at the forefront of studies is ideally needed for developing a successful therapy. Certain key elements are relevant to these principles, including measurement of SN for diagnosis and evaluating outcomes (“who”), prism strength/presence of aftereffects, treatment intensity, arm visibility, and activities during prism adaptation (“how”). The impacts of these elements on intervention effects in SN are reviewed below.

### Key elements of prism adaptation treatment

#### Defining SN and measuring outcomes

The fact that there is no gold standard for SN measurement (39, 40) is reflected in the PAT literature where various SN tests and criteria have been used as the operational definition of SN diagnosis and outcome measures in different studies. While BIT-c and CBS are commonly used for outcome measures, studies differ in screening criteria, which may or may not involve BIT-c or CBS. Some studies excluded patients with mild SN (101–103) to potentially ensure the detectability of therapeutic effects after PAT. However, it is unknown what level of SN severity is likely to benefit from PAT, which is a critical question for enhancing treatment potency. In addition, using BIT-c or CBS to determine SN severity is not sufficient to capture the heterogeneity of SN. A variety of subtypes exist, as briefly reviewed at the beginning of this article, in terms of reference frames (egocentric vs. allocentric), regions of space based on the distance from the examinees' body (personal, peri-personal, and extra-personal space), or whether a symptom presentation primarily depends on perceptual input or motor output. Few PAT studies have focused on whether certain symptoms or subtypes would derive differential benefits from PAT and thus enhance treatment effects [cf. (104)].

Measurement of PAT outcomes requires that the instruments used are reliable and sensitive to any treatment effects. Thus, the heterogeneity of SN also has an impact in terms of matching tests to symptoms as they may not have the same responsiveness to changes in certain patients. In addition, ceiling effects (BIT-c) or floor effects (CBS) of common scales need to be considered. Some studies also add measures less directly related to SN *per se* to explore to what extent PAT may have beneficial impacts on rehabilitation outcomes and other aspects of patients' lives. Studies have assessed abilities to perform daily tasks (105, 106), for example, using the Functional Independence Measure (FIM) (107), and estimated the likelihood of returning home after PAT (86). However, very few studies have investigated other outcome measures indicative of fall risk, quality of life, community reintegration, employment, and other milestones of brain injury rehabilitation. While the choice of outcome measures may be related to the purpose of the study, a lack of consensus can make developing general principles difficult.

#### Prism strength

Prism strength is measured in diopter, and the amount of visual shift, measured in visual angle degree, is also commonly reported. Prism lenses used in PAT are worn by individuals with SN during a session guided by a therapist and removed after the session, and it is important to note that prism strength is *not* tailored to individual patients. Thus, regardless of SN severity, all patients wear lenses with the same prism strength in a given PAT study. This one-size-fits-all approach is rather different from other clinical approaches using prescribed prisms to correct visual field cuts, double vision, or other visual impairments. In these cases, the prescriptions are used to personalize the visual assistance needed during all waking hours. In the context of PAT, prisms are only used to shift the visual field during a short period of time (e.g., 10–20 min) to induce sensorimotor adaptation and aftereffects, and aftereffects can fade away in minutes to hours after prism removal. To our knowledge, the question of personalization of prisms in PAT related to SN severity has not yet been addressed.

The importance of prism strength is related to the detectability of a therapeutic effect. While there is yet no systematic study examining the relationship between prism strength and the size of PAT therapeutic effects, a relationship can be inferred across studies with different prism strengths. Two randomized sham-controlled studies using 10-diopter prism lenses (inducing 6° visual shift) (108) or 5° shifting prism lenses (109) did not find PAT effects on the BIT-c or CBS. Studies using higher prism strength with at least a 10° shift showed positive, albeit mixed, results (see later sections of the article). Prism strength determines the detectability of prism aftereffects. The size of an aftereffect is correlated with, but usually less than, the prism strength, although there can be much individual variation. Tasks to measure prism aftereffects are usually done without visual feedback to prevent de-adaptation, including pointing straight ahead with eyes closed or pointing at visual targets with eyes open, but with the vision of the limb completely occluded. The latter is often referred to as open-loop pointing. Facchin et al. (110) showed that the open-loop pointing aftereffect was approximately 40% of the prism-induced visual shift. That is, while a set of 20-diopter prism goggles shifts the visual field by 11.4° horizontally to one side (non-neglected side) during prism adaptation, the aftereffect is only on average 4.5° to the other side (neglected side) of space. This size of aftereffect is in contrast to a 1° aftereffect reported after only 10-diopter (5.7°) prisms were used (108).

The inclusion of aftereffect screening may be important as patients who do not demonstrate measurable aftereffects are unlikely to benefit from PAT, as undetectable aftereffects may suggest the basic neural mechanism underlying sensorimotor adaptation is impaired. Prism adaptation requires the involvement of the cerebellum and the visual, motor, and parietal cortices (111, 112). If this fundamental neural mechanism is impaired and aftereffects are not generated, then the therapeutic effects based on changes to related brain areas are unlikely to be detected (measured using neuropsychological tests and ecological assessments) (76). Some studies and treatment protocols, but not all, thus exclude patients whose prism aftereffects fail to meet a certain criterion (49, 113–115). For example, in the KF-PAT protocol (49) used in several studies and clinical practices (82, 83, 88, 101, 102, 116), aftereffects are measured using open-loop pointing and straight-ahead pointing with eyes closed. In this protocol, an aftereffect is defined when an after-adaptation performance is more toward the neglected side of space in comparison to the before-adaptation performance. If patients do not demonstrate aftereffects in either pointing measure, in the first three consecutive sessions, continuing PAT is not recommended. This variability in aftereffect generation and the need for measurement can be seen in a study by Ten Brink et al. (115), who measured the aftereffect after PAT with open-loop pointing by asking patients to point to a central visual target presented briefly in front of them before closing their eyes and pointing. Patients who did not show an aftereffect greater than 3 cm toward the neglected side of space after the adaptation procedure (100 pointing movements while wearing prisms) were required to repeat the adaptation procedure with prisms with 50 more pointing movements. Twelve (35%) of the participants in the treatment group failed to reach the 3 cm criterion in more than 50% of the sessions. Thus, there is a need to measure aftereffects and their relations to PAT therapeutic effects, and yet the presence or the extent of an aftereffect has been variably measured and used in different studies, making comparison across studies more difficult.

#### Arm visibility and adaptation activities when wearing prisms

The extent of visibility of arm reaching movements while wearing prisms may be important to the size of aftereffects (117) or therapeutic effects (118), although the research is inconsistent (110), due, at least in part, to what aftereffect or outcome measure is used. There are two major approaches related to arm visibility—terminal and concurrent exposure. Terminal exposure restricts vision of the reaching arm and only allows patients to see a few centimeters of the final movement trajectory as the fingertip gets close to the target. Concurrent exposure allows patients to see the latter one-third to half of the arm movement (part of the forearm, hand, and finger) or the entire limb reaching toward the target or during functional activities. In both approaches, immediate visual feedback of the final performance is available to participants.

A small number of studies compared terminal exposure and concurrent exposure directly in individuals with SN while the concurrent-exposure condition differed in the amount of limb visible among the studies. Facchin et al. (119) used a within-participant study design where participants were given either terminal or concurrent exposure to an 11.3° visual shift in a counterbalanced order in one session. Concurrent exposure allowed almost the entire limb visible while pointing to targets. No difference was seen in the size of open-loop pointing aftereffects or in the results of neuropsychological tests for SN (119). Two other studies compared the two procedures with multi-session PAT administration using prism lenses inducing 10° of visual shift: Ladavas et al. (118) compared terminal exposure, concurrent exposure (pointing toward a visual target with the latter half of arm movement being visible), and no prism adaptation in three groups of participants, who completed 10 PAT sessions over 2 weeks, in a randomized controlled trial (RCT). Ladavas et al. found no difference in open-loop pointing aftereffects between the two exposure conditions, but a greater reduction of SN symptoms of impairment and behavioral performance on standard SN tests in the terminal exposure group compared to the concurrent exposure group. Thus, while Ladavas et al. could not draw a direct link between differences in aftereffects and therapeutic effects, terminal exposure resulted in greater therapeutic effects than concurrent exposure. Fortis et al. (120) used a randomized, crossover study design where one procedure was administered for 10 sessions (2 sessions a day) within a week, followed by the other procedure. Fortis et al.'s procedure for concurrent exposure was integrated with functional activities that required arm-reaching movements toward visual targets (e.g., coin collection and card sorting). Fortis et al. found no difference in the results of SN tests at the impairment or functional level and general functional improvement (120). Thus, the small but diverse literature has not determined whether and how the beneficial effects of PAT are associated with the extent to which arm movements are visible to patients during adaptation.

In terms of activities performed when wearing prisms, a finger pointing task to simple targets, e.g., dots or lines, either with terminal or concurrent exposure, is the most common task reported in the literature following Rossetti et al. (67) Variations to this approach do exist, however. As described above, Fortis et al. (120) used a variety of functional reaching tasks during concurrent exposure. Other activities include using a pen to mark targets on paper (102, 121), a digital stylus to reach targets on a touch screen (115, 122), and a controller, represented as a virtual fingertip, to reach targets in immersive virtual reality (123). When in an MRI scanner, various setups have been used with patients for finger pointing (124, 125) or imagined pointing (126). All different activities, except for the imagined pointing, follow the same principle—the arm reaching toward a visible target followed by immediate visual feedback, repeatedly for 50–100 times per PAT session. Methods can differ, however, in terms of task, visual angle of workspace, and number of targets or responses. Whether any of these methods are more effective than the others in terms of inducing aftereffects or PAT therapeutic effects remains mostly untested.

#### Treatment intensity

Few prospective studies have been conducted to directly investigate what number and frequency of PAT sessions are sufficient to result in short-term SN reduction or long-term functional improvement. Nonetheless, the literature provides some insights. While most RCTs have followed the once-daily 2-week treatment regimen (e.g., 10 sessions in total), Goedert et al. (127) conducted a secondary analysis of an RCT and found 4–6 sessions over 2 weeks might be as sufficient as 10 sessions over 2 weeks for lasting effects shown on BIT-c. In contrast, Rode et al. (70) using a low treatment intensity of one session a week over 4 weeks (four sessions in total) showed no PAT effects on BIT-c or FIM, suggesting treatment intensity can be an important factor.

The importance of treatment intensity may be related to the underlying neural mechanisms of prism adaptation. During prism adaptation and measurement of aftereffects, circuits between the cerebellum and motor cortex are activated, and bilateral parietal cortices and several parts of the intact hemisphere are involved as well (66, 112, 126). A working hypothesis proposes that after repeated and frequent sessions, functional connectivity between the parietal cortex and other parts of the brain may be formed, leading to symptom changes with lasting effects beyond eye–hand coordination and visuomotor behaviors (66, 76). Therefore, in line with the evidence cited above, theoretically, the number and frequency of PAT sessions matter and deserve more investigation.

### Studies included in the recent meta-analyses

*None* of the recent meta-analyses of prospective RCTs (53, 79–81), when selecting studies through systematic reviews, considered key elements of PAT—prism strength, arm visibility and activity when wearing prisms, and treatment intensity—except for outcome measures. Longley et al. (53) included RCTs that measured functional abilities as outcomes. Qiu et al. (80) included RCTs that used BIT-c or CBS as an outcome measure. Li et al. (79) included RCTs that used BIT-c, CBS, FIM, star cancellation test, line bisection, or reading as an outcome measure. Szekely et al. (81) included studies with any control condition and an outcome measure that was CBS, BIT-c, or a cancellation test. However, circumstances of when, why, and for whom are critical for effective behavioral intervention development according to the NIH Stage Model.

To develop a broader perspective of PAT studies and how the variability of research might affect the four recent meta-analyses (53, 79–81), we categorized studies that were included in those meta-analyses based on the NIH Stage Model. We used the stage descriptions in the NIH Stage Model (87) as guidelines and operationalized each stage for the context of PAT clinical research focused on SN rehabilitation (Table 1). A total of 22 studies were included in the four recent meta-analyses (53, 79–81). For the purpose of the exercise, we extracted information from 20 of those studies that were written in English and that examined PAT therapeutic effects by comparing PAT to a control condition. Therefore, one study (128) reviewed by Szekely et al. (81) was excluded because it was not written in English. Another study (129) included in Longley et al. (53) was also excluded because there was no control condition but two comparative conditions using different experimental treatments.

Among the 20 studies, 4 studies (105, 108, 113, 115) were included in all recent systematic reviews. Three studies (70, 102, 109) were included in any three reviews. Three studies (118, 130, 131) were included in any two reviews. Ten studies (67, 101, 114, 116, 132–137) were only included in Szekely et al. (81). Each author of the present article independently reviewed the full-text publications of these 20 studies. Stage labels were determined after multiple group meetings following the operational definitions of the NIH Stage Model for PAT research (Table 1). As summarized in Table 2, three studies (67, 134, 136) were categorized as Stage 0. There were eight Stage 1 studies (70, 108, 109, 116, 118, 130, 135, 137) and five Stage 2 studies (102, 113, 114, 131, 133). One study (132) was categorized as Stage 1 or 2 because it was designed like a Stage 2 study but the analysis did not compare outcomes between the PAT and control groups, which made the study fall into Stage 1 for method feasibility. Three RCTs (101, 105, 115) met the Stage 3 criteria. Within each stage category, studies differed in SN diagnosis, time post incident at baseline, age, prism strength, limb visibility when wearing prisms, treatment intensity in terms of the total number of sessions and frequency of sessions, and outcome measures (Table 2). Thus, the studies were varied within each development stage and even more different across stages.

**Table 2:** Studies categorized as Stage 0, 1, 2, and 3 based on the operational definition of the NIH Stage Model for Behavioral Intervention Development.

Study (included in which prior meta-analysis)^a^	Study design	Injured hemisphere (cause)	SN inclusion criterion	Control	Total sample size at baseline	Time post incident at baseline or first PAT session (mean or median)	Age in years (mean or median)	Prism-induced visual shift (prism lens)	Limb visibility when wearing prisms (description or quote from paper)	Activity during PA	Tx intensity	Outcome measures used before and after tx
Stage 0												
Rossetti et al. (67) (S)	Randomized, sham-controlled	Right (stroke)	Unspecified	Sham	12	9 weeks (mean)	62 (mean)	10° (wedged lens with diopter unspecified)	Terminal (only the fingertip visible at the end of movement)	50 times of finger pointing to visual targets	1 session	Schenkenberg line bisection; Albert line cancellation; scene copying; daisy drawing; text reading
Rousseaux et al. (136) (S)	Crossover (order counterbalanced)	Right (stroke)	Detected in ≥2 of 3 tests (line bisection, Bells Test, and scene copying)	Sham	18	54.3 days (mean)	55.5 (mean)	10° (lens unspecified)	Unknown (“a mask prevented subjects from viewing their arm”)	50 times of finger pointing to visual targets	1 session per condition	Bells Test; line bisection; Ogden scene drawing; single-word reading; single-non-word reading; text reading
Facchin et al. (134) (S)	Crossover (order randomized)	Right (stroke)	Detected in ≥1 of 4 tests (line bisection, star cancellation, sentence reading, and comb and razor)	Sham	21	174 days (mean)	68 (mean)	16.7° (30-diopter Fresnel lens)	Terminal (only the fingertip visible at the end of movement)	90 times of finger pointing to visual targets	1 daily session (10 total) over 2 weeks	Star cancellation; line bisection; sentence reading; comb and razor; standardized neurological examination; electrocutaneous stimulation
Stage 1												
Serino et al. (130) (Li, S)	Controlled	Right (stroke)	BIT-c < 129/146 or BIT-b < 67/81	Usual care	24	13.2 months (mean)	66.8 (mean)	10° (lens unspecified)	Terminal (only the fingertip visible at the end of movement)	90 times of finger pointing to visual targets	1 daily session (10 total) over 2 weeks	BIT; single-word reading
Turton et al. (108) (Li, Lo, Q, S)	Randomized, sham-controlled	Right (stroke)	Detected in line bisection or star cancellation	Sham	34	46.1 days (mean)	71.5 (mean)	6° (10-diopter wedged lens)	Terminal (only the fingertip visible at the end of movement)	90 times of finger pointing	1 daily session (10 total) over 2 weeks	CBS; BIT-c
Vangkilde & Habekost (137) (S)	Randomized controlled	Right (various types of brain injuries)	Detected in ≥2 of 7 tests (line bisection, visual extinction, Mesulam Stars Cancellation, Mesulam Letters Cancellation, article reading, Baking Tray, and ROCF)	Usual care	11	28.6 months (mean)	57 (mean)	10° (15-diopter Fresnel lens)	Terminal (only the fingertip visible at the end of movement)	90 times of finger pointing to visual targets	2 daily sessions (20 total) over 2 weeks	Line bisection; Mesulam star cancellation; Mesulam letter cancellation; figure copying; Baking Tray; Where's Wally Test; cupboard test; self-reported function
Ladavas et al. (118) (Q, S)	Randomized, sham-controlled	Right (stroke)	BIT-c < 129/146 or BIT-b < 67/81	Sham	30	7 months (mean)	63.2 (mean)	10° (lens unspecified)	Terminal vs. Concurrent (limb invisible for the first 1/2 of movement)	90 times of finger pointing to visual targets	1 daily session (10 total) over 2 weeks	Single-word and non-word reading; BIT
Mancuso et al. (109) (Li, Lo, S)	Randomized (by site), sham-controlled	Right (stroke)	Detected in ≥2 of 7 tests (Bells Test, line orientation, BIT's line crossing, line bisection, figure copying, object finding, and dealing cards)	Sham	22	159.2 days (mean)	67 (mean)	5° (lens unspecified)	Terminal (only the fingertip visible at the end of movement)	90 times of finger pointing to visual targets	1 daily session (5 total) over 5 days	Line cancellation; Bells Test; line orientation; BIT's line bisection, figure copying, object finding, and dealing playing cards
Rode et al. (70) (Li, Lo, Q)	Randomized, sham-controlled	Right (stroke)	Detected in some of the four tests (line bisection, Balloons Test, figure copying, and text reading)	Sham	18	51.6 days (mean)	58.5 (mean)	10° (wedged lens with unspecified diopter)	Concurrent (limb invisible for the first 1/3 to 1/2 of movement)	80 times of finger pointing to visual targets	1 daily session (4 total) over 4 weeks	FIM; BIT
Hreha et al. (116) (S)	Retrospectively controlled	Right (stroke)	CBS (KF-NAP) > 1/30	Usual care	26	16.5 days (mean)	78.3 (mean)	11.4° (20-diopter wedged lens)	Concurrent (limb invisible for the first 1/2 to 2/3 of movement)	60 times of using a pen to make a mark through a circle or horizontal line (or up to 20 min)	1 daily session (10 total) over 2 weeks	CBS (KF-NAP); BIT's star cancellation and line bisection; motor FIM
Longley et al. (135) (S)	Randomized, controlled, 3:1 stratified group allocation	No criteria (stroke)	Four-point scale^b^	Usual care	43	15 days (median)	69 (mean)	12.5° (25-diopter wedged lens)	Terminal (only the fingertip visible at the end of movement)	90 times of finger pointing (or up to 5 min)	1 daily session (up to 15 total) over 3 weeks	Hearts cancellation test; star cancellation; reading test; CBS (KF-NAP)
Stage 1 or 2												
Frassinetti et al. (132) (S)	Randomized (by site), controlled	Right (stroke)	BIT-c < 129/146 or BIT-b < 67/81	Usual care	13	9.4 months (mean)	61.7 (mean)	10° (wedged lens with unspecified diopter)	Terminal (only the fingertip visible at the end of movement)	90 times of finger pointing	2 daily sessions (20 total) over 2 weeks	BIT; Bells Test; word and non-word reading; fluff test; room description test; object reaching test
Stage 2												
Nys et al. (113) (Li, Lo, Q, S)	Randomized, sham-controlled	Right (stroke)	Detected in ≥2 of 4 tests (BIT's line bisection, star cancellation, figure copying, and drawing)	Sham	16	9.7 days (mean)	62.8 (mean)	10° (wedged lens with unspecified diopter)	Concurrent (limb invisible at the beginning of movement)	100 times of finger pointing	1 daily session (4 total) over 4 days	Schenkenberg line bisection; scene copying; letter cancellation; BIT
Serino et al. (131) (Li, S)	Randomized, sham-controlled	Right (stroke)	Mean of BIT-c and BIT-b < 97/114	Sham	20	7.3 months (mean)	61.6 (mean)	10° (lens unspecified)	Terminal (only the fingertip visible at the end of movement)	90 times of finger pointing	1 daily session (10 total) over 2 weeks	BIT; Bells Test; single-word and non-word reading
Mancuso et al. (133) (S)	Randomized, sham-controlled	Right (stroke)	Unspecified	Sham	40	60 days (mean)	65.5 (mean)	10° (lens unspecified)	Terminal (only the fingertip visible at the end of movement)	90 times of finger pointing	2 daily sessions (20 total) over 2 weeks	Bells Test; BIT-c; CBS
Vaes et al. (114) (S)	Randomized, sham-controlled	Right (stroke)	Detected in line bisection or star cancellation	Sham	43	50.3 days (median)	63.3 (median)	10° (wedged lens with unspecified diopter)	Terminal (only the fingertip visible at the end of movement)	80 times of finger pointing	1 or 2 daily sessions (7 total) over 7–12 days	Bells Test; diamond cancellation; Schenkenberg line bisection; rectangle bisection; search time test; butterfly and clock copying; extinction test; spatial memory test; maze navigation
Goedert et al. (102) (Lo, Q, S)	Randomized, controlled	Right (stroke)	BIT-c < 129/146	Usual care	17	43.7 days (mean)	63.8 (mean)	11.4° (20-diopter wedged lens)	Concurrent (limb invisible for the first 1/2 to 2/3 of movement)	60 times of using a pen to make a mark through a circle or horizontal line (or up to 20 min)	1 daily session (10 total) over 2 weeks	BIT; CBS (KF-NAP)
Stage 3												
Mizuno et al. (105) (Li, Lo, Q, S)	Randomized, sham-controlled	Right (stroke)	Detected in ≥1 BIT-c test	Sham	38	65.7 days (mean)	66.3 (mean)	12° (Fresnel lens with unspecified diopter)	Terminal (only the fingertip visible at the end of movement)	90 times of finger pointing	2 daily sessions (20 total) over 2 weeks	BIT; CBS; FIM; stroke impairment assessment set
Ten Brink et al. (115) (Li, Lo, Q, S)	Randomized, sham-controlled	No criteria (stroke)	Detected in ≥1 of 3 tests (line bisection, shape cancellation, and CBS)	Sham	69	39.3 days (median)	60.4 (median)	10° (lens unspecified)	Unknown (“a board was held under the chin to prevent viewing of the hand at its starting position but allowing an unobstructed view of the targets and terminal errors”)	100 times of using a digital stylus to point and touch a visual target on a touch-screen tablet	1 daily session (10 total) over 2 weeks	CBS; MAC asymmetry; shape cancellation
Vilimovsky et al. (101) (S)	Randomized, sham-controlled	No criteria (stroke and TBI)	CBS (KF-NAP) > 10/30	Sham	23	73 days (median)	54.8 (median)	11.4° (20-diopter wedged lens)	Concurrent (limb invisible for the first 1/2 to 2/3 of movement)	60 times of using a pen to make a mark through a circle or horizontal line (or up to 20 min)	1 daily session (10 total) over 2 weeks	CBS (KF-NAP); Bells Test, line bisection; scene copying

Within each stage, studies were presented from upper to lower rows by the year published and in alphabetical order of the first author's name.

BI, brain injury; BIT-b, behavioral subtests of the behavioral inattention test; BIT-c, conventional subtests of the behavioral inattention test; CBS, Catherine Bergego scale; KF-NAP, Kessler Foundation neglect assessment process; MAC, mobility assessment course; PAT, prism adaptation treatment; RCT, randomized controlled trial; ROCF, Rey-O figure copying; SN, spatial neglect; TBI, traumatic brain injury; tx, treatment.

^a^ The initial (or first two letters if sharing the same initial) of the first author's last name of the meta-analysis is noted: Li for Li et al. 2021; Lo for Longley et al., 2021; Q for Qiu et al., 2021; S for Szekely et al., 2023.

^b^ Longley et al. (135) screened patients with “a 4-point scale (none, mild, moderate, severe) using a combination of functional observations and [occupational therapists’] clinical judgement.”

For example, the three studies categorized as Stage 3 (i.e., “real-world efficacy” trials) were all sham-controlled RCTs; however, experimental groups received a variety of PAT with different prism strength but potentially comparable (10–12° visual field shift), different arm reach activities (finger pointing to visual targets vs. hand-holding devices to touch or mark visual stimuli) with different extents of limb visibility during prism adaptation, and different treatment intensity (20 sessions over 2 weeks, 10 sessions over 2 weeks). While Mizuno et al. (105) integrated PAT as part of the regular practice, Ten Brink et al. (115) and Vilimovsky et al. (101) had patients complete PAT as an additional treatment on top of regular therapy. What was considered regular could vary considerably given that these RCTs were conducted in three different countries where rehabilitation care services are provided differently. Regarding participant selection, all the RCTs included stroke survivors, and Vilimovsky et al. (101) included two individuals with traumatic brain injury (one in each group). Mizuno et al. (105) included patients with right brain damage and excluded patients with left brain damage, while Ten Brink et al. (115) and Vilimovsky et al. (101) included individuals based on SN diagnosis regardless of which hemisphere was damaged (Table 2). In terms of participants' age and time post-brain injury, Mizuno et al.'s (105) cohort appeared older than the others, and Vilimovsky et al.'s (101) sample may have received PAT later than the others' time post-incident. In short, although these three RCTs met the Stage 3 criteria, they were qualitatively diverse, reflecting the lack of consensus regarding SN diagnosis and treatment methods with PAT.

Thus, unsurprisingly, there was considerable heterogeneity of studies (Table 2) included in recently published meta-analyses (53, 79–81), resulting in null or some effects. In other words, sometimes PAT works and sometimes it does not, without providing the circumstances of when and why PAT works and for whom. However, circumstances of when, why, and for whom are critical for effective behavioral intervention development according to the NIH Stage Model.

## Discussion

### Circumstances (development stages) matter

High heterogeneity is a major limitation of meta-analyses that have been conducted in recent years (53, 79–81). Szekely et al. (81), for example, recognized the difficulty in combining the results of various studies and the small numbers of included studies in each of their meta-analyses, which impeded the efforts to include variables as candidate moderators. Szekely et al. (81) suggested that one solution may be the standardization of treatment protocols and outcome measures. However, other factors contribute to the high heterogeneity as revealed in our categorization exercise guided through the NIH Stage Model (Table 2). One of Szekely et al.'s meta-analyses was focused on three RCTs, led by Turton (108), Mizuno (105), and Ten Brink (115) (order by year published), because they met the highest quality standard of research conduct determined by the authors (81) and used the same outcome measure, i.e., CBS. The standard, however, did not take treatment apparatus, procedure, or intensity into account. One of the studies (108), which used low-strength prism lenses (shifting visual field by 6°), was categorized as Stage 1 because it contributed to the understanding of PAT and enabled the suggestion for using high-strength prism lenses that shift visual field by at least 10°. The other two studies, by Mizuno et al. (105) and Ten Brink et al. (115), were categorized as Stage 3. Pulling studies at different development stages contributes to the heterogeneity of data sources.

Lunven et al. (138) promptly responded after the publication of Szekely et al. and suggested that the null effect resulting from Szekely et al.'s analyses may have been washed out because the RCTs included in the meta-analyses did not consider individual differences in lesioned brain areas or impaired brain connectivity, which may have played significant roles in mediating PAT effects (66, 121, 126). This critique can be expanded to another factor that may affect PAT therapeutic effects and thus a meta-analysis result, which is the timing of PAT provision relative to time post-brain damage because brain connectivity changes over time (7, 139). No RCT has yet prospectively triaged patients based on their profiles regarding SN symptoms or brain lesions while it is well known that SN is heterogenous. Thus, more studies are required.

### More research is needed before standardizing treatment protocols

While much has been learned about PAT since Rossetti et al.'s groundbreaking study (67), it is not nearly enough to result in any standardized best practice recommendations for the use of PAT as an SN therapy. To put it in the framework of the NIH Stage Model (Figure 1a), treatment refinement and modification studies (Stage 1) combined with efficacy testing (Stage 2) and neural mechanism exploration based on individual patients' brain connectivity profiles (all stages) are needed to determine a treatment protocol appropriate for a specific cohort of patients with SN. In addition, it is essential to involve the intended users (i.e., clinicians and management leaders in rehabilitation care services) in determining a treatment protocol that is not only scientifically sound but also practically feasible, which is one of the lessons learned from the implementation project by Hreha et al. (88).

### More research is needed before standardizing outcome measures

As reviewed above and summarized in Table 2, CBS and BIT-c are relatively popular among PAT studies, but it does not necessarily mean that CBS or BIT-c has been used following the same methods in different studies or either is ideal for measuring outcomes.

Standardization of the administration and scoring of outcome measures is critical when combining studies in a meta-analysis, and it is unclear whether this is always accomplished. Regarding the administration of CBS, for example, all three studies categorized as Stage 3 used CBS as an outcome measure. Vilimovsky et al. (101) followed KF-NAP, and per protocol, patients were assessed by an occupational therapist with each session completed during one single visit. The two other studies followed the CBS questionnaire format. Mizuno et al. (105) did not specify how, or by what discipline, the assessment was administered, while Ten Brink et al. (115) stated that nurses, occupational therapists, and physical therapists shared the responsibility. In terms of scoring, the final CBS score is a prorated score (to account for unrated items), rather than the total score (simple sum of rated items), which is the method used to quantify SN severity regardless of how the CBS is administered. Unrated items are usually due to patients’ cognitive or motor impairment (92). However, many studies, such as Mizuno et al. (105), did not specify whether the prorated score was used as the outcome measure.

The large variability in SN can affect how well an outcome measure aligns with symptoms and their changes. The BIT-c is limited in capturing a range of changes in SN symptoms partly because it is confined in the peri-personal space defined by a regular letter-sized or A4 paper. In addition, the BIT-c total score is heavily weighted on cancellation tests, accounting for 89% of the total score of 146 (46) or 72% of the total score of 181 (with more scoring details for figure copying and representational drawings) (47). While CBS is more sensitive than many neuropsychological or non-ecological assessments such as BIT-c (44, 140), it cannot capture SN symptoms beyond the 10 items during other daily activities such as reading, wayfinding in unfamiliar environments, medication management activities, and crossing streets. Thus, CBS may be insensitive to long-term changes in SN as patients regain function over months or years. Studies are required to develop ecological outcome measures sensitive to chronic SN before conducting future PAT trials focused on long-term effects. More efforts are needed to identify and standardize an outcome measure or test battery for future RCTs evaluating SN treatment efficacy and effectiveness.

### More research is needed before conducting another meta-analysis

PAT is not ready for meta-analysis focused on clinical efficacy, based on the NIH Stage Model for Behavioral Intervention Development. This conclusion is derived from the fact that the optimal combination of key elements that define “who” should be included and “how” it works in PAT are not yet determined. In other words, knowledge gaps exist in every key element of PAT that should be included in efficacy and effectiveness research. There is no consensus on “who” (e.g., defining SN and outcome measures and taking into account individual patient heterogeneity such as brain lesion profile, age, and time post-stroke) or “how” (e.g., developing consensus on PAT protocols, including prism strength, arm visibility, and treatment intensity). Only when a PAT protocol is ready with all the key elements validated to be contributing to therapeutic effects, should the protocol be examined for clinical efficacy. While there are standardized PAT protocols published by different research groups, there is no consensus. This is a major contributor to a lack of consistent findings from recent meta-analyses, and clinicians should interpret meta-analysis evidence very cautiously.

The practice-based evidence of PAT is encouraging (82–86), based on retrospective analyses of clinical data, and can potentially be categorized as Stage 4 (Table 1). While those studies do not answer all the questions regarding PAT effectiveness for the same reasons stated above for prospective studies, findings can inform the designs and planning of new prospective studies to further refine certain key elements.

## Conclusion

Research provides guidance and helps clinicians evaluate whether a treatment, such as PAT, may be beneficial to patients. The current knowledge established in the literature provides a general direction for prism strength (at least 20 diopters or shifting visual field by at least 10°) insights into treatment intensity (at least three sessions per week and at least four sessions in total) and suggested the use of prism aftereffects for screening for PAT eligibility. Repeated (50–100) visuomotor activities that require arm movement toward a visible target are necessary when wearing prisms, but arm visibility (terminal vs. concurrent exposure) and types of visuomotor activities need further research to determine how they contribute to PAT therapeutic effects.

No project, to our knowledge, has been planned to examine different strategies to implement PAT clinically. Few cognitive rehabilitative therapies have ever reached Stage 5, and most established interventions and treatments are adopted by certain clinical practices and implemented organically. We advocate for more research and implementation projects involving rehabilitation practitioners, using the NIH principles of developing behavioral interventions as a path toward generating real-world evidence for clinical effectiveness, guiding the clinical use of PAT.
